# Instruments That Predict Oncology Treatment Risk in the Senior Population

**Published:** 2017-07-01

**Authors:** Jessica K. Schiefen, Lydia T. Madsen, Joyce E. Dains

**Affiliations:** The University of Texas, MD Anderson Cancer Center, Houston, Texas

By 2030, an estimated one out of five Americans will be over 65 years of age. An expanding senior population will profoundly impact the health-care system related to cancer care (Centers for Disease Control and Prevention, 2013). Cancer is both a disease associated with aging and the leading cause of death among men and women aged 60 to 79 years old (Kim & Hurria, 2013). Treating the senior adult with cancer has specific, unique challenges. Physiologic changes associated with aging lead to decline in organ function, impacting outcomes with treatment. Health status and functional reserve also vary significantly in senior adults, making chronologic age alone an unreliable predictor of the risk for treatment complications (Korc-Grodzicki, Holmes, & Shahrokni, 2015).

The National Comprehensive Cancer Network (NCCN) classifies senior patients into three categories: young-old (ages 65 to 75 years), old (ages 76 to 85 years), and oldest-of-old (over age 85 years). The use of common chemotherapy regimens in young-old adults with good performance status is supported by clinical trials. However, few studies address either old patients, oldest-of-old patients, or those with poor performance status (NCCN, 2016), leaving limited guidance for determining the risk of chemotherapy toxicity in this senior population (Baitar et al., 2014).

## PRETREATMENT SENIOR ASSESSMENT TOOLS

National Comprehensive Cancer Network (2016) Guidelines recommend a comprehensive geriatric assessment (CGA) in the senior population to assess life expectancy and the risk of morbidity. The CGA is defined as "a multidimensional, interdisciplinary diagnostic process focusing on determining an older person’s medical, psychosocial, and functional capabilities to develop a coordinated and integrated plan for treatment and long-term follow-up" (Wildiers et al., 2014, p 2595). A CGA assesses functional status, comorbidities, polypharmacy, nutritional status, cognitive function, psychological status, socioeconomic issues, and geriatric syndromes (NCCN, 2016). Although a CGA may help detect geriatric syndromes, predict toxicity with treatment, improve treatment, and identify when supportive interventions are indicated (NCCN, 2016), it is not routinely used in oncology practice. Its limited use is presumed to be associated with both increased time and resource administration requirements (Korc-Grodzicki et al., 2015; NCCN, 2016).

Recent research has focused on evaluation of the predictive value of instruments to effectively assess potential toxicity in senior patients prior to chemotherapy treatment. Current data suggest that two instruments are clinically efficient in determining patients at higher risk for chemotherapy toxicity (Extermann et al., 2012; Hurria et al., 2011; Luciani et al., 2015; Nie, Liu, Li, & Bai, 2013). This review, which will compare the Cancer and Aging Research Group (CARG) chemotherapy toxicity calculator and the Chemotherapy Risk Assessment Scale for High-Age Patients (CRASH) score, is intended to provide guidance to the oncology practitioner in clinical practice on efficient, validated instruments that assess risk for chemotherapy toxicity in senior adults with cancer. 

**Cancer and Aging Research Group Chemotherapy Toxicity Tool **

Hurria et al. (2011) developed the CARG tool from data obtained through a prospective multicenter study involving 500 cancer patients, aged 65 years and older. A prechemotherapy assessment was completed and patients were followed throughout the chemotherapy course to capture grade 3 through grade 5 toxicities (Hurria et al., 2011). 

*Risk Stratification:* The CARG tool uses both objective and subjective data from 11 risk factors associated with an increased risk for chemotherapy toxicities (Table 1; Hurria et al., 2011). Subjective information is completed by the patient, with the remaining objective information extracted from the chart by a health-care professional. Each risk factor is assigned a score (Table 1) and when scores are totaled, patients are categorically stratified as low risk (0 to 5 points), intermediate risk (6 to 9 points), and high risk (10 to 19 points; Table 2; Hurria et al., 2011). 

**Table 1:**
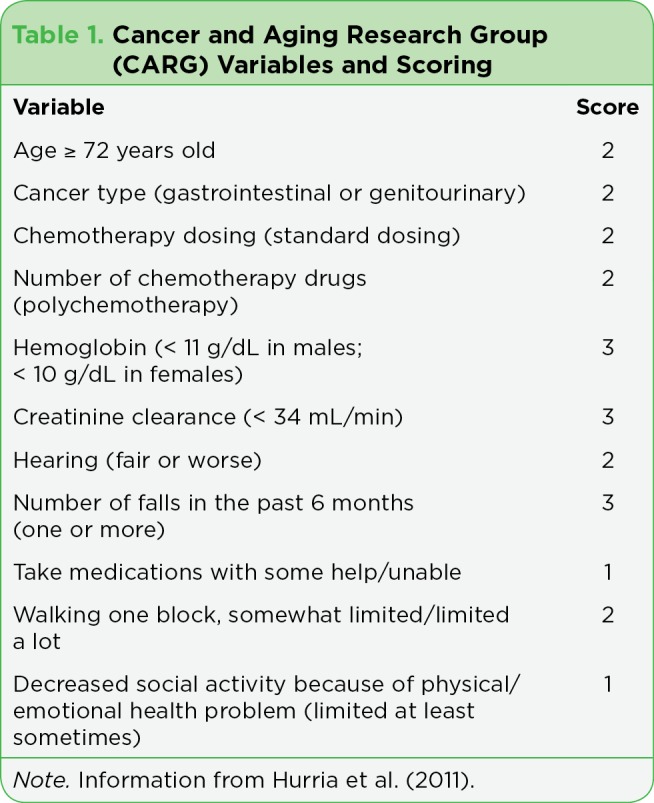
Cancer and Aging Research Group (CARG) Variables and Scoring

**Table 2:**
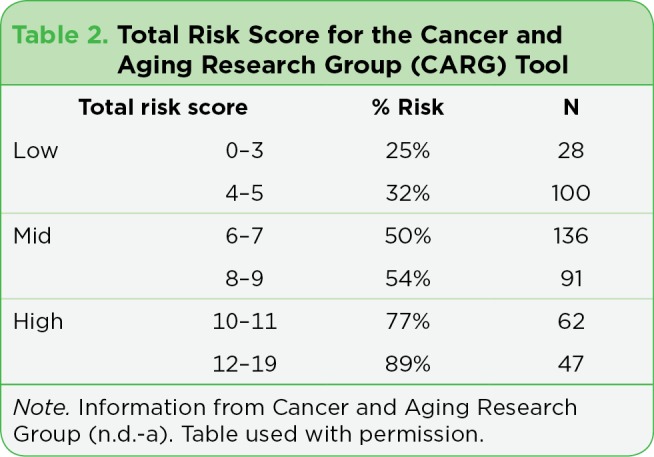
Total Risk Score for the Cancer and Aging Research Group (CARG) Tool

*Administration Considerations:* Medical staff must input data for the four objective questions regarding laboratory values and treatment planning. The remaining seven questions on physical function and emotional concerns are answered by the patient. Scores are totaled and categorized; results are interpreted by the clinician. An interactive, electronic version of the CARG tool is available online (CARG, n.d.-a).

*Psychometrics:* This tool was internally validated in the study by Hurria et al. (2011) and independently validated by Nei et al. (2013). Nie et al. (2013) completed a study with 120 lung cancer patients, aged 65 years and older, scheduled to receive a new chemotherapy regimen. Prior to treatment, patients completed a questionnaire and laboratory work. Each patient’s chemotherapy course was reviewed to identify grade 3 to 5 chemotherapy toxicities (Nie et al., 2013). The NCCN (2016) Guidelines recommend additional validation studies. 

*Predictive Value:* Hurria et al. (2011) and Nie et al. (2013) found that the CARG tool was able to significantly predict chemotherapy toxicity (*p* < .001; *p* < .001) and to discriminate risk for chemotherapy better than the Karnofsky Performance Score (*p* = .19; *p* = .322). 

*Limitations of the Studies:* The Hurria et al. (2011) study included patients with different tumor types and treatment regimens with the intent of determining risk factors common to geriatric oncology patients. However, other risk factors that predict toxicity based on tumor type or regimen may exist (Hurria et al., 2011). Nie et al. (2013) assessed a homogeneous cancer population of lung cancer patients in China, although the tumor type was not a determined risk factor. The NCCN (2016) Guidelines recommend further validation studies for the CARG tool. It is important for future validation studies to be conducted among both homogeneous and heterogeneous populations to identify specific variables pertinent to certain cancers and treatment. 

The CARG tool was developed from the study conducted by Hurria et al. (2011), in which the association between chemotherapy toxicity and a variety of variables was assessed. Although polychemotherapy was not statistically significant in this cohort (*p* = .2422), this variable is included, as aging is associated with both decreased bone marrow reserve and an increase in myelosuppressive complications from chemotherapy (Hurria et al., 2011). This finding is further supported by Luciani et al. (2015), in which polychemotherapy was found to be predictive of hematologic toxicities on univariate (*p* = .003) and multivariate analyses (*p* < .001; Hurria et al., 2011).

In contrast, statistically significant variables were not included in the CARG tool. For example, "timed get up and go" score (*p* = .0446) and body mass index (BMI; *p* = .0136) are significantly associated with the development of chemotherapy toxicity yet are not included in the CARG tool (Hurria et al., 2011). It is unclear why these two variables were omitted from the tool. 

The CARG tool requires patient self-reporting of hearing impairment, number of falls in the past 6 months, ability to manage home medications, limitations in walking a block, and decreased social activity due to physical or emotional health. Self-reported data may not accurately depict deficits; senior patients may be unaware of deficits due to gradual adaption of a limitation or may have concerns disclosing limitations that may impact treatment planning. Input from caregivers, family members, and clinicians may provide an additional and more complete assessment of physical function and mental state. 

A critique of the two studies evaluating the CARG tool (Extermann et al., 2012; Nie et al., 2013) noted the inconsistency of the cutoff scoring for risk stratification. Nie et al. (2013) scores ranged from 0 to 14, and Hurria et al. (2011) scores ranged from 0 to 19, indicating a patient whose total score was 5 is at low risk according to the cutoff set by Hurria et al. (2011). However, a score of 5 in the Nie et al. (2013) study was considered mid risk for chemotherapy toxicity. Nie et al. (2013) did not account for tumor type (gastrointestinal vs. genitourinary) in the total score, as the study sample included lung cancer patients only, which may account for the scoring differences. 

**Chemotherapy Risk Assessment Scale for High-Age Patients (CRASH) Score**

The CRASH score was developed by Extermann et al. (2012) to stratify the risk for chemotherapy toxicity. Extermann et al. (2012) conducted a study with 518 patients, aged 70 years old and older, starting chemotherapy. Patients had a baseline assessment prior to starting chemotherapy. Toxicity was followed with weekly laboratory values and medical evaluations at the beginning of each cycle and at the end of treatment. Patients were followed up to 1 month after the completion of chemotherapy (Extermann et al., 2012). 

*Risk Stratification:* The CRASH score is an 8-item assessment that integrates both patient variables and chemotherapy regimen. Chemotherapy regimens are given a numerical value from 0 to 2 based on their potential for chemotherapy toxicities. This is called a Chemotox score. This score is calculated using the MAX2 index, which is a measure of the overall risk for severe toxicity with certain chemotherapy regimens (Extermann et al., 2012). The CRASH score divides toxicity risk into hematologic and nonhematologic scores. Increased risk predictors for toxicity are outlined in Table 3 (Extermann et al., 2012).

**Table 3:**
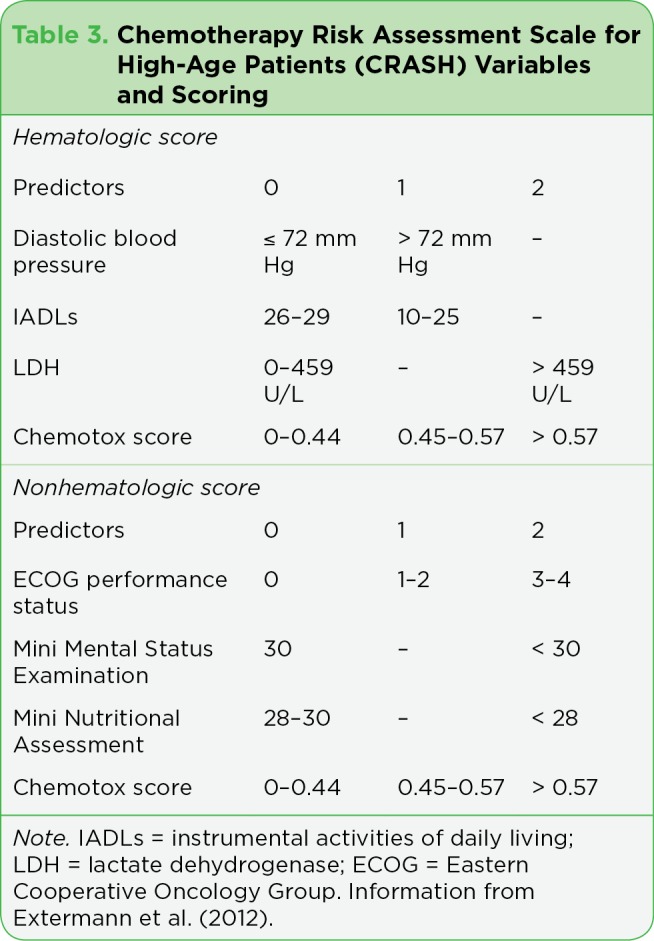
Chemotherapy Risk Assessment Scale for High-Age Patients (CRASH) Variables and Scoring

Each variable is given a score ranging from 0 to 2 (Table 3). Scores are totaled, with patients stratified into four categories: low, medium-low, medium-high, and high (Extermann et al., 2012). A score of 0 to 3 is considered low risk for the development of chemotherapy toxicity, 4 to 6 is considered medium-low risk, 7 to 9 is considered medium-high risk, and 9 or above is considered high risk (Table 4; Moffitt Cancer Center, n.d.). 

**Table 4:**
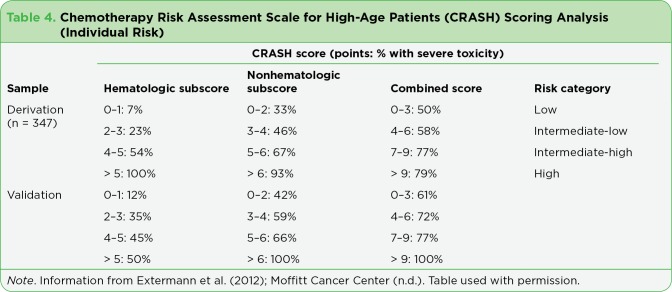
Chemotherapy Risk Assessment Scale for High-Age Patients (CRASH) Scoring Analysis (Individual Risk)

*Administration Considerations:* This tool is completed by a health-care provider by entering diastolic blood pressure, laboratory data (lactate dehydrogenase [LDH]), performance status, instrumental, chemotoxicity score, instrumental activities of daily living (IADL), and Mini Mental Status Examination (MMSE) and Mini Nutritional Assessment (MNA) assessments. An interactive, electronic version of the CRASH calculator is available online (Moffitt Cancer Center, n.d.).

*Psychometrics:* The CRASH score provides an integrated risk score that was stable over two validations (Extermann et al., 2012). National Comprehensive Cancer Care (2016) Guidelines recommend additional validation of the CRASH score. 

*Predictive Value:* A total of 64% of patients in the study by Extermann et al. (2012) experienced toxicities. The CRASH score was significantly associated with predicting hematologic (*p* = .005) and nonhematologic toxicities (*p* < .05; Extermann et al., 2012). 

*Study Limitations:* As with the CARG tool, the CRASH score included variables not significant for the development of chemotherapy toxicity. For example, creatinine clearance is included yet is not significantly correlated with either hematologic or nonhematologic toxicities (*p* = .74, *p* = .09; Extermann et al., 2012). However, renal function is a standard baseline assessment prior to chemotherapy initiation, so the authors presume that dosing and regimen were adjusted for decreased creatinine clearance prior to CRASH assessment. 

The cancer type was not stratified, making risk factors associated with specific cancers and treatments unavailable (Hurria et al., 2011). No published results on additional studies within a homogeneous cancer population are available at this time.

The CRASH score also incorporates a Chemotox score to stratify the risk for toxicity from specific chemotherapy regimens. The Chemotox score is calculated using the MAX2 index (Extermann et al., 2012). Currently, there are 45 different chemotherapy treatment plans that have been converted from MAX2 score to Chemotox scores. There is an equation available online to calculate a MAX2 and Chemotox score (Moffitt Cancer Center, n.d.). This extra step requires additional time and resources, which may be cumbersome for the clinician. 

In addition to calculating a Chemotox score, providers complete additional assessments such as the IADL, MMSE, and MNA (Extermann et al., 2012). To obtain a CRASH score, a modified 9-item IADL instrument is required, which is derived from Lawton’s standard 8-item questionnaire (Lawton, 1988). Completing these additional assessments and calculating the Chemotox score take additional time and resources to complete. 

The CRASH score, like the CARG tool, requires the use of patient self-reporting. The CRASH score uses assessments that require patients to report functional limitations, nutritional deficits, and mental health status (Extermann et al., 2012). Self-reporting may not accurately depict deficits, and/or patients may underreport limitations.

## DISCUSSION AND IMPLICATIONS

Cancer is a disease of the aging, and as the senior population continues to grow, oncology practitioners will treat an increasing number of senior patients. Currently, the CGA is the gold standard of practice when it comes to assessing risk and mortality among older patients (NCCN, 2016). By using the CGA, advance practice providers (APPs) can predict severe treatment-related toxicity and overall survival (Wildiers et al., 2014). However, the CGA can be time-consuming and requires additional resources. Oncology practitioners can utilize shorter instruments to determine which patients are at risk for chemotherapy toxicities. Current data suggest that the CARG tool and the CRASH score are efficient and valid instruments for determining which patients may be at a higher risk for chemotherapy toxicity (Extermann et al., 2012; Hurria et al., 2011; Luciani et al., 2015; Nie et al., 2013) by using risk stratification of high, medium, or low based on patient variables, treatment regimens, and cancer type.

Both tools have an online calculator, which makes computing total scores less cumbersome (CARG, n.d.-a; Moffitt Cancer Center, n.d.). However, input on the online calculator for the CRASH score requires the completion of MMSE, MNA, and IADL as additional geriatric assessments, requiring time and additional resources for completion. 

These instruments provide an opportunity to standardize the risk for chemotherapy toxicity by stratifying patients into risk groups based on personal characteristics and treatment variables that increase risk for the development of chemotherapy toxicities (Extermann et al., 2012; Hurria et al., 2011; Nie et al., 2013). These brief but objective assessment results may allow for communication between the patient and the clinician to discuss goals of treatment, such as palliative vs. standard dosing. Making patients aware of their individual risk for the development of toxicities with chemotherapy may assist them with treatment discussions. 

Currently, the CARG tool has been validated in an additional study, with ongoing trials taking place (CARG, n.d.-b; Nie et al., 2013). The CARG tool was developed from a study of participants with a variety of cancers and further studied by Nie et al. (2013) among a homogeneous population of lung cancer patients. Based on its ability to stratify patients into risk groups, its feasibility, ease of use, and predictive value among both a heterogeneous and homogeneous cancer population, the CARG tool is the most efficient and predictive tool currently available to assess risk for chemotherapy toxicity (Hurria et al., 2011). 

**Recommendations for Future Research**

Further studies are needed to validate the use of short instruments in predicting the risk for chemotherapy toxicity among the senior population (NCCN, 2016), as there remain limited data available on these tools and their predictive capabilities. Additionally, future studies should examine these instruments in disease-specific senior populations to identify specific risk factors for that cancer type (Baitar et al., 2014; Extermann et al., 2012; Hurria et al., 2011; Luciani et al., 2015).

## CONCLUSION

Seniors are a need-specific cohort within the cancer patient population. To adequately assess the risk for chemotherapy, a clinically time-efficient tool designed to predict risk for treatment-related toxicities is needed to provide guidance for appropriate treatment regimens in the senior cancer population. Although historically performance status has been used to determine the ability to tolerate chemotherapy in the senior population, its effectiveness remains unclear (Extermann et al., 2012; Hamaker et al., 2014; Hurria et al., 2011; Luciani et al., 2015; Nie et al., 2013). The CGA can help predict severe treatment-related toxicities; however, time and resource requirements limit its routine use. The CARG tool and the CRASH score are time-efficient tools that can be utilized in practice to assess the critical factors found in the CGA (Extermann et al., 2012; Hurria et al., 2011; Nie et al., 2013). Additional studies are needed to further validate and establish these instruments’ utility in clinical practice.
